# Catheterless Long-Term Ambulatory Urodynamic Measurement Using a Novel Three-Device System

**DOI:** 10.1371/journal.pone.0096280

**Published:** 2014-05-19

**Authors:** Sebastian Wille, Pauline Schumacher, Jenny Paas, Dirk Tenholte, Okyaz Eminaga, Ute Müller, Noemi Muthen, Jan Mehner, Oliver Cornely, Udo Engelmann

**Affiliations:** 1 Department of Urology, University of Cologne, Cologne, Germany; 2 ZKS Köln-Zentrum für Klinische Studien, Cologne, Germany; 3 Chemnitz University of Technology, Faculty of Electrical Engineering and Information Technology, Chemnitz, Germany; 4 BMP Labor für medizinische Materialprüfung GmbH, Aachen, Germany; 5 Cologne Excellence Cluster on Cellular Stress Responses in Aging-Associated Diseases (CECAD), Medical Faculty, University of Cologne, Cologne, Germany; Instituto de Engenharia Biomédica, University of Porto, Portugal

## Abstract

**Aims:**

Long-term urodynamics are required because bladder-emptying disorders are often not clearly revealed by conventional urodynamics. Patients with severe clinical overactive bladder symptoms, for instance, often show normal results. This may be due to the short evaluation time and psychological factors that complicate conventional urodynamics. This study aimed to develop an ambulatory three-component urodynamic measurement system that is easy to operate, registers urodynamic parameters for several days, and has no negative impact on the patient.

**Methods:**

We developed an intravesical capsule combined with a hand-held device to register voiding desire and micturition, and an alarm pad device that detects urine loss. Recently, the intravesical capsule and its proven function were detailed in the literature. Here, we present detailed *in vitro* results using a female bladder model. The flexible capsule was C-shaped to minimize the risk of expulsion from the bladder during micturition. Results of biocompatibility evaluation of the intravesical capsule, which is called Wille Capsule (WiCa) are described.

**Results:**

The WiCa with an oval nose and a maximum outer diameter of 5.5 mm was easily inserted through a 25-French cystoscope. Removing the WiCa by grasping the nose using the female model with bladder was easily conducted. Expulsion of the WiCa during voiding was avoided through a novel C-shaped device design. Based on *in vitro* cytotoxicity studies, the capsule is a promising and safe device.

**Conclusion:**

Our novel system is an innovative minimally-invasive tool for accurate long-term urodynamic measurement, and does not require inserting a transurethral catheter.

## Introduction

The need for long-term urodynamics is justified because a clear diagnosis often cannot be made by conventional clinical urodynamics in patients with bladder emptying disorders. For instance, in patients suffering from overactive bladder (OAB) [1], conventional urodynamics show normal results in approximately 45% of OAB cases without detrusor-overactivity (DO) [2]. In the series by Hashim et al, the rate of OAB patients without DOs was 58% [3]. This might be due to the short time schedule allotted to conventional urodynamics, with an average clinical investigation time of 20 to 40 minutes. Other explanations for underdiagnosing OAB include psychological reasons due to the rather disconcerting conditions of urodynamic testing, and also by the inability of a 40-minute office-visit investigation to reflect typical daily conditions.

Catheterless pressure measurement is of a great interest for many medical applications including intracranial pressure measurement and real-time blood pressure measurement [4] Some devices have been designed especially for intravesical pressure measurement [4]–[6].

Implantable measurement systems have to be very small, so device power supply is one of the most crucial parameters. If no continuous measurement is desired or the system has to remain in the body for more than a few days, telemetric power supplies are applicable. In most cases, this is achieved by inductive power transfer, and measured data are also transmitted wirelessly. The main disadvantage of these systems is that they need at least one easily accessible extracorporeal port, which limits patient daily activities.

We recently published details on the intravesical capsule [7] and demonstrated size, technical construction, maximal operation time, sensor sensitivity, and accuracy. In this paper we present detailed *in vitro* results using a model female bladder. The flexible capsule is now C-shaped to minimize the risk of expulsion from the bladder during micturition. Results from biocompatibility testing of the intravesical capsule, which is called Wille Capsule (WiCa), are also shown.

## Materials and Methods

Development of the three urodynamic devices was performed in cooperation with the Chemnitz University of Technology, Faculty of Electrical Engineering and Information Technology, Chemnitz, Germany. This project was supported by the Center for Clinical Trials Cologne (ZKS Köln) and by the German Federal Ministry of Research and Education (BMBF grant 01KN1106). After the feasibility analyses were completed in 2009, the project was started according to International Conference on Harmonisation-Good Manufacturing Clinical Practice standards as a Medical Product Law study [8]. A patent has been applied for this device system including the C-shaped intravesical capsule, hand device, alarm pad device (Deutsches Patent-und Markenamt), DE 10 2011 014 220A1.

### Capsule Design, Fixation, and Removal

**Figure 1:**
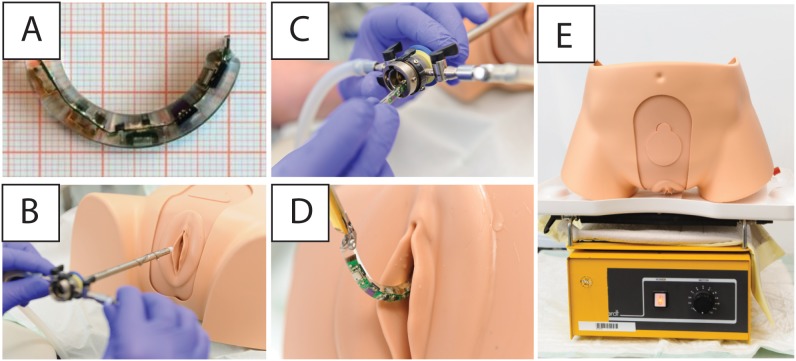
The components of the WiCa system. A: C-shaped Wille-Capsule (WiCa). B: *In vitro* testing for the WiCa using a bladder model. C: Insertion was straightforward using a standard 25 French cystoscope. D: The WiCa could be easily grasped and removed using forceps. E: Bladder model on a vibrating plate.

Requirements for the system include easy handling for the patient with minimal daily activity impairment, and the ability to classify clearly bladder-emptying disorders through continuous urodynamic measurements lasting 1 to 3 days. The flexible circuit board is encapsulated with the batteries in biocompatible silicone (silicone elastomer MED-6015 Part A + B by NuSil Silicone Technology, California). The flexible capsule is C-shaped to minimize the risk of expulsion from the bladder during micturition. In the straightened state (length, 45 mm; diameter, 5.5 mm), the capsule can be pushed or pulled through a cystoscope for bladder insertion/removal. Inside the bladder, the flexible capsule reverts to the C-shaped state, which prevents expulsion from the bladder. Figure 1A shows the C-shaped WiCa with its nose structure designed to facilitate capsule removal. This shape is generated during silicone molding. A bladder model (Company Limbs & Things, USA) was used to simulate insertion and removal of the WiCa (Figure 1 B). A 25-French (Figure 1C) cystoscopic tube (Olympus) was utilized to insert the WiCa. The capsule was removed using endoscopic forceps (Olympus) (Figure 1D).

The bladder model was both vertically and horizontally fixed on a vibrating plate (Company Gerhardt, LS 10), level 3 for 24 hours during vibration (Figure 1E) to test whether the capsel remains in the bladder.

### Hand Device

**Figure 2:**
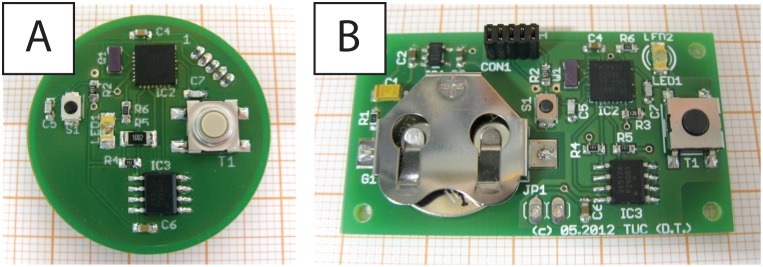
Electronic circuits. A: The handheld device. B: The alarm pad.

The handheld device was designed to provide a digital voiding diary synchronized to the pressure measurement capsule and alarm pad. Similar to the measurement capsule, the handheld device consists of a 2.5 V power supply, a microcontroller (MSP430 by Texas Instruments) and an EEPROM for storing the time stamps of desire to void and micturition, which can be distinguished because they are specifically marked. Device size is not as crucial as for the sensor capsule, so we use a 3 V lithium coin cell (Type CR1620) for its power supply. To extend battery life, the voltage is reduced to 2.5 V by a linear regulator (TPS77025). The electronics (Fig. 2A) are soldered onto a circular board intended to be encapsulated by a watch-like case.

### Alarm Pad

To detect all urine loss events, an alarm pad was constructed. The alarm pad is the third component of the system and registers all urine loss. The electronic concept of the alarm pad is analogous to alarm pads used in treating enuresis patients. Special electronics, similar to the hand-held device, consist of a power supply, microcontroller, and an EEPROM. The alarm pad electronics are shown in Figure 2B.

### Data Transfer and Analyses

The initial idea was to provide urodynamic measurement for as long as possible. We therefore did not choose the use of online data transfer, to reduce power requirements. Consequently, the capsule was equipped with an USB-interface. Stored data are transferred by a wire connector after removing and opening the capsule.

### Cytotoxicity Investigations of the WiCa

#### Culture and investigation conditions

Biocompatibility of any medical device is prerequisite for its acceptance for clinical use [10]. Cytotoxicity tests, as a part of the biocompatibility testing, were performed in the accredited laboratories of BMP GmbH (Laboratory for medical material testing, Aachen, Germany), according to German Institute for Standardization (Deutsches Institut for Normung, DIN), (European Norm, EN) ISO 10993-5 standards. Both cytotoxicity tests were performed according to DIN EN ISO 10093-5∶2009. L929 murine fibroblasts (CCL 1, NCTC clone 929, National Collection of Type Cultures) were cultured in Eagle’s Minimum Essential Medium (EMEM) supplemented with 10% fetal bovine serum and penicillin/streptomycin solution (10 IU/ml, and 100 µg/ml, respective final concentrations) at 37°C in a humidified atmosphere of 5% CO_2_ and 95% air.

#### Metabolic activity assays

The metabolic activity of the murine cells was evaluated using colorimetric quantification of conversion of the tetrazolium salt XTT by mitochondrial dehydrogenases (Cell Proliferation Kit II (Roche Diagnostics GmbH, Mannheim, Germany). The assay is based on the ability of metabolically active cells to reduce the tetrazolium salt XTT to orange-colored formazan compounds (Cell Proliferation Kit II, Roche Diagnostics GmbH, Mannheim, Germany).

After 72 hours, media conditioning with the capsule, murine fibroblasts were incubated for 24 hours with the resulting capsule-conditioned extract. Extraction medium (EMEM/1% penicillin/streptomycin) not conditioned by capsule contact was served as a negative control and sodium dodecyl sulfate, 20 µg/ml in extraction media, was used as a positive control. Two independent experiments each in 8 replicates were performed using capsule-conditioned media, and negative and positive controls. Cellular mitochondrial activity was determined in 8-fold dilutions of conditioned media or controls, using the Cell Proliferation Kit II. Formazan production was evaluated on a microplate reader using a 450 nm optical filter with a 690 nm reference wavelength.

#### Membrane integrity assessment

Membrane integrity was assessed by a double-staining method using fluorescein diacetate and ethidium bromide (EtBr). Fluorescein diacetate easily permeates the cell membrane and is enzymatically converted by intracellular esterases into a green fluorescent anionic product that is retained intracellularly. EtBr only enters cells with disrupted membrane integrity, where it intercalates with DNA and forms a red fluorescent complex. Detection of vital cells was visualized using a fluorescence light microscope (Model DMLB; Leica, Mikrosystem Vertriebs GmbH, Germany) with filter system I3, damaged cells were detected using a filter system N2.1. Commercial PTFE foil (bioFOLIE 25, In-vitro Systems and Services GmbH, Göttingen, Germany) and polyvinylchloride foil (PVC 7500, Rehau AG & co. KG, Rehau, Germany) were used as negative and positive controls respectively. Two independent experiments were performed using capsule and negative and positive controls. The surface of capsule as well as the surface of negative and positive controls were brought in direct contact with L929 fibroblast cells und incubated for 24 hours at 37°C in a humidified atmosphere of 5% CO_2_, 95% air.

## Results

### Capsule Function

The main requirements regarding the capsule were small size and adequate power supply for long-term urodynamic measurement. *In vitro* capsule tests were successful. External magnet system activation occurred without problems. The prototype length was approximately 45 mm and diameter was 5.5 mm. The WiCa was easily inserted through the 25-French (Figure 1C) cystoscopic tube. After the WiCa was manually inserted into the tube the obturator of the cystoscope (Olympus) was used to push the WiCa into the bladder where the flexible capsule reverts to the C-shaped state. *In vitro* studies using a female bladder model verified the straightforward removal of the WiCa using a 25 French cystoscope by grasping the nose of the capsule with forceps (Figure 1D). Application of lubricating gel or sterile water simplifies advancement of the capsule through the cystoscope. It was clearly demonstrated that the WiCa remained in the bladder model using a vibrating plate (Company Gerhardt, LS 10), level 3 for 24 hours. This was tested both ways: the bladder model vertically and horizontally fixed (Figure 1E).

**Figure 3:**
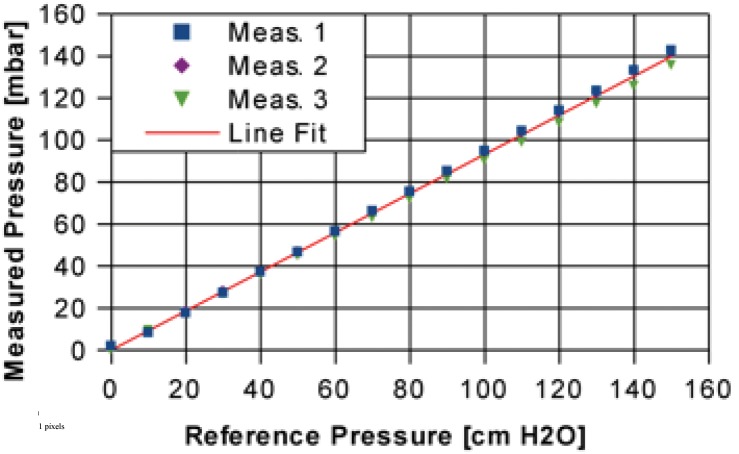
Pressure sensitivity of the pressure measurement capsule. (Meas. = Measurement, lin fit =  linear fit).

We tested the pressure-signal of the molded sensor at a range of 0 to 160 cm H_2_O overpressure (Fig. 3) using a water-filled pipe made of acrylic glass. The molding of the flexible circuit board slightly decreases the pressure sensor sensitivity. Therefore, the measured pressure does not exactly correspond to the reference pressure but is 0.93× the reference pressure. The measurement deviation depends on the pressure; at 160 cm H_2_O we observed a maximum deviation of less than 0.1% of the actual reading.

### In vitro Analyses

#### Metabolic activity

Cellular metabolic activity was quantified by the XTT assay. The optical density of the negative control was standardized at 100% and compared with the relative values of the material samples and the positive control. Capsule-conditioned media (72 hours) did not inhibit mitochondrial activity when subsequently applied to cultured L-929 fibroblasts for 24 hours, with formazan production measured at approximately 99% of levels observed in negative controls. This indicates that no cytotoxic compounds leaked from the device during the testing period. According to grading criteria DIN EN ISO 10993-5∶2009, these data are indicative of no capsule-related cytotoxicity.

#### Membrane integrity

**Figure 4:**
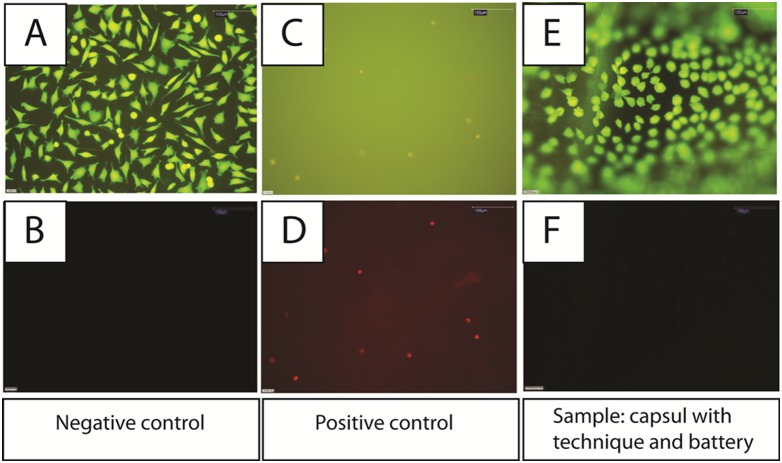
Viability of murine L929 fibroblasts incubated for 24 hours on the surface of negative control (PTFE foil). Fluorescein diacetate was taken up by cells and converted to a green fluorescent product, which was retained intracellularly, indicating maintained cell viability (4A). Ethidium bromide was included in the cell suspension on the surface of the negative control but was not taken up by cells, demonstrating intact functional cell membranes (4B). Viability of murine L929 fibroblasts incubated for 24 hours on the surface of the positive control (PVC). Fluorescein diacetate uptake and conversion to a fluorescent product was hindered versus the negative control, indicating compromised cell viability (4C). Ethidium bromide included to the cell suspension on the surface of the positive control was taken up by cells and intercalated with DNA, staining the nuclei red, demonstrating compromised cell membrane functionality (4D). Viability of murine L929 fibroblasts which were brought in direct contact with surface of the intravesical capsule device containing electronics and batteries for 24 hours. Fluorescein diacetate was taken up by cells and converted to a green fluorescent product, which was retained intracellularly, indicating cell viability (4E). Ethidium bromide included in the cell suspension on the surface of the intravesical capsule was not taken up by cells, demonstrating intact functional cell membranes (4F). All photomicrographs were taken at 200× original magnification.

We used grading criteria DIN EN ISO 10993-5∶2009 to assess cytotoxicity of capsule-conditioned EMEM on cultured L929 fibroblasts. After a 24-hour incubation period in the unconditioned negative control media, there was no alteration of cellular membrane integrity. The cytoplasm was uniformly stained green by fluorescein diacetate (Fig. 4A) and there was no indication of EtBr entry into the cells (Fig. 4B). Cells incubated for 24 hours in the sodium dodecyl sulfate-containing positive control media had disturbed membrane integrity, evidenced by lack of fluorescein diacetate retention in the cytoplasm and clear EtBr staining of nuclei (Fig. 4C and 4D). Importantly, incubation of L929 cells with capsule-conditioned media did not disrupt cellular membrane integrity, evidenced by positive cytoplasmic fluorescein staining and a lack of EtBr nuclear staining that was indistinguishable from the negative control-treated cells (Fig. 4E and F). Thus, the capsule/battery device does not release cytotoxic compounds into culture media at human body temperatures, through ≥3 days of exposure.

## Discussion

Urodynamics have become an important means of diagnosing bladder storage and emptying disorders over the last 40 years. However, patients with severe symptoms often have normal results in conventional urodynamic testing and the underlying etiology might be multifactorial [7]. To overcome this diagnostic limitation, we developed a minimally invasive, catheterless system for long-term urodynamic evaluation that consists of the intravesical capsule, a hand-held device, and an alarm pad. The new measurement capsule makes possible non-traumatic capsule insertion into the patient's bladder and allows for prolonged (≥72 hrs) outpatient urologic measurement, while the patients perform their normal daily routine.

An important aspect of intravesical measurement systems is a stable sensor device fixation within the bladder to ensure that the system will not be expelled during micturition. Clasbrummel and Muhr [5] suggested suturing the system inside the bladder; other systems are large enough that expulsion is impossible, but they are not installable through a cystoscope. Some authors have not provided any information how to prevent an expulsion [9]. We designed a C-shaped flexible capsule that, when temporarily straightened, can be pushed or pulled through a cystoscope. Once placed inside the bladder, the device regains its C-shape and cannot be expelled. This could be clearly shown by bladder model testing for 24 hours with the model both vertically and horizontally fixed on the vibrating plate. However, any pressure changes due to abdominal stress like micturition or coughing could not be fully mimiced with this kind of model. Therefore, further in vivo studies are needed to show whether the capsule will remain in the bladder under normal life condition.

Urgency can be recorded through the hand device, urine loss is detected by the alarm pad, and DOs are recorded by the capsule, all simultaneously.

Widespread use of this urodynamic measurement system will improve urologic therapy for many patients. For example, OAB patients could be more specifically diagnosed, and thus treated in a more specific way. The key question in OAB patients is still whether or not DO occurs during an urge or urge incontinence episode. Without long-term urodynamics with simultaneous recording of urgency and urine loss, this cannot currently be conclusively determined. We assume that DO occurs more often than it is diagnosed, and is associated with urge or urge incontinence.

The strong association between DO and OAB signs was also recognized by Radley et al, who correlated questionnaires regarding urgency with DO prevalence in AUM (ambulatory urodynamic measurement) [11]. DO is presumably a requirement for the successful therapy using anti-cholinergic (anti-ACh) treatments. We assume that anti-ACh administration in the absence of DO might not have the same effect as if incontinence occurs in the presence of DO. Assessing DO with long-term urodynamics could result in higher accuracy of anti-ACh therapeutic application. In addition, excluding DO in potential non-responders would avoid anti-ACh side effects such as mouth dryness, accommodation disorder, and deterioration of visual acuity. These patients could instead be more successfully treated with other primary therapies, e.g., neuromodulation, electromotive drug administration, or botulinum toxin A.

The rapid development of silicone-coated devices has benefited a variety of fields in biology and medicine. Nonetheless, to insert this silicone-coated device into the human body, it must be proven to be biologically compatible with tissues [12]. However, safety evaluations of silicone-sheathed devices have rarely been performed in the urological field.

According to internationally accepted guidelines and recommendations for biological evaluation of medical devices: DIN EN ISO 10993-1∶2010, DIN EN ISO 10993-5∶2009, and DIN EN ISO 10993-12∶2009, *in vitro* investigations of the capsule and battery showed no device-related cytotoxicity [13], [14] Cultured L929 fibroblasts retained normal metabolic activity, viability, and uncompromised membrane integrity after culture in capsule-conditioned media [15]. Therefore, the capsule used in this study is definitively non-cytotoxic. Additional investigations recommended in DIN EN ISO 10993-1∶2010 on the fields of chemical characterization for extractable substances and leachable metallic ions and the recommended evaluation of biological risk potential are currently being performed.

## Conclusion

We have developed a unique, catheterless, nontoxic, long-term, ambulatory, urodynamics measurement system that includes an intravesical capsule, a hand-held device, and an alarm pad unit. This project represents trend-setting innovation and advancement for accurately and minimally invasively diagnosing urinary incontinence pathophysiology.
